# S-nitrosoglutathione reductases are low-copy number, cysteine-rich proteins in plants that control multiple developmental and defense responses in Arabidopsis

**DOI:** 10.3389/fpls.2013.00430

**Published:** 2013-11-05

**Authors:** Shengbao Xu, Damian Guerra, Ung Lee, Elizabeth Vierling

**Affiliations:** ^1^Department of Biochemistry and Molecular Biology, University of Massachusetts-Amherst, Amherst, MA, USA; ^2^School of Life Sciences, Lanzhou University, Gansu, China; ^3^Department of Biochemistry and Molecular Biophysics, University of Arizona, Tucson, AZ, USA

**Keywords:** S-nitrosoglutathione (GSNO), S-nitrosoglutathione reductase (GSNOR), nitrosative stress, trichomes, nitric oxide homeostasis, formaldehyde metabolism, glutaredoxin, pathogen defense

## Abstract

S-nitrosoglutathione reductase (GSNOR) is believed to modulate effects of reactive oxygen and nitrogen species through catabolism of S-nitrosoglutathione (GSNO). We combined bioinformatics of plant GSNOR genes, localization of GSNOR in *Arabidopsis thaliana*, and microarray analysis of a GSNOR null mutant to gain insights into the function and regulation of this critical enzyme in nitric oxide (NO) homeostasis. GSNOR-encoding genes are known to have high homology across diverse eukaryotic taxa, but contributions of specific conserved residues have not been assessed. With bioinformatics and structural modeling, we show that plant GSNORs likely localize to the cytosol, contain conserved, solvent-accessible cysteines, and tend to be encoded by a single gene. *Arabidopsis thaliana* homozygous for *GSNOR* loss-of-function alleles exhibited defects in stem and trichome branching, and complementation with Green fluorescent protein (GFP) -tagged GSNOR under control of the native promoter quantitatively rescued these phenotypes. GSNOR-GFP showed fluorescence throughout Arabidopsis seedlings, consistent with ubiquitous expression of the protein, but with especially high fluorescence in the root tip, apical meristem, and flowers. At the cellular level we observed cytosolic and nuclear fluorescence, with exclusion from the nucleolus. Microarray analysis identified 99 up- and 170 down-regulated genes (≥2-fold; *p* ≤ 0.01) in a GSNOR null mutant compared to wild type. Six members of the plant specific, ROXY glutaredoxins and three BHLH transcription factors involved in iron homeostasis were strongly upregulated, supporting a role for GSNOR in redox and iron metabolism. One third of downregulated genes are linked to pathogen resistance, providing further basis for the reported pathogen sensitivity of GSNOR null mutants. Together, these findings indicate GSNOR regulates multiple developmental and metabolic programs in plants and offer insight into putative routes of post-translational GSNOR regulation.

## Introduction

In plants, biological processes ranging from leaf stomatal closure to auxin perception in roots and pathogen infection involve nitric oxide (NO) (Neill et al., 2002; Floryszak-Wieczorek et al., 2007; Lozano-Juste and Leon, 2011; Terrile et al., 2012). While NO itself is ostensibly active, it is also thought to be transmitted to distal targets via low molecular weight S-nitrosothiols (SNOs), of which the glutathione (GSH) adduct S-nitrosoglutathione (GSNO) is the most abundant (Broniowska et al., 2013; Corpas et al., 2013). GSNO can profoundly affect protein activity through glutathionylation and nitrosation of cysteines (Romeo et al., 2002; Giustarini et al., 2005; Zaffagnini et al., 2013), implying that cells require mechanisms to spatiotemporally control GSNO levels. Catabolism of GSNO by S-nitrosoglutathione reductase (GSNOR) is common to eukaryotes and many bacteria and is believed to be responsible for this regulation of GSNO levels (Liu et al., 2001; Staab et al., 2008). GSNOR exhibits NAD/H-dependent oxidoreductase activity toward a broad spectrum of aliphatic compounds, but its preferred substrates are GSNO and S-hydroxymethylglutathione (HMGSH), an intermediate in formaldehyde metabolism (Jensen et al., 1998; Achkor et al., 2003; Kubienová et al., 2013). While GSNO catabolism has been observed with Cu-Zn superoxide dismutase, GSH peroxidase, xanthine oxidase, and human carbonyl reductase 1 (CR1), the former three enzymes merely regenerate NO [summarized in Broniowska et al. (2013)], and residues critical to interaction between CR1 and GSH adducts are not conserved in plants (Bateman et al., 2008). GSNOR is therefore considered the primary catalyst for GSNO catabolism in plants.

The importance of GSNOR to plant growth, development and stress responses has been highlighted by several studies. Lowered *GSNOR* expression in *Arabidopsis thaliana* (Arabidopsis), resulting from a null mutation (*atgsnor1-3/hot5-2*) or RNAi, was correlated with higher SNO content and differential susceptibilities to pathogens (Feechan et al., 2005; Rustérucci et al., 2007). The effect of absence of GSNOR was extended by Lee et al. (2008) who described a thermotolerance defect that was rescued with NO scavengers. Other phenotypes of plants with GSNOR mutations include diminished fertility and resistance to programmed cell death induced by paraquat, an herbicide that elicits robust reactive oxygen species (ROS) production (Lee et al., 2008; Chen et al., 2009). These concomitant gains and losses of function are analogous to consequences of GSNOR inhibition in mammals, for which both enhanced carcinogenesis and abated severity of inflammatory diseases are observed (Wei et al., 2010; Sun et al., 2011; Tang et al., 2013). Such pleiotropy suggests GSNOR participates in both homeostatic maintenance and biotic and abiotic stress responses.

The evolutionary conservation of GSNOR is high (Liu et al., 2001), and although the consequences of *GSNOR* depletion have been described at the organismal level for Arabidopsis (Lee et al., 2008; Chen et al., 2009; Kwon et al., 2012), a molecular etiology for the *GSNOR* loss-of-function phenotype is lacking. Here we sought to address the issues of how GSNOR activity could be regulated and of what processes are impacted by changes in GSNO levels and, therefore, potentially regulated by nitrosation or glutathionylation of protein effectors. We searched for conserved and unique features of plant GSNOR proteins, localized GSNOR at the tissue and cellular levels, and measured global changes in the transcriptome of an *atgsnor/hot5* null mutant in Arabidopsis. Our data demonstrate that most sequenced green plant genomes are predicted to encode a single copy of GSNOR characterized by a high content of positionally-conserved cysteines. GSNOR is found in the cytosol and nucleus throughout the plant, and is thus available to modulate GSNO concentration in most if not all cells. Moreover, alterations in the transcriptome of Arabidopsis homozygous for the *atgsnor1-3*/*hot5-2* null allele exhibited dysregulated expression of pathogen response and calcium signaling genes, but higher expression of a subset of glutaredoxin (GRX)-encoding genes. Together, these data suggest GSNOR facilitates multiple homeostatic and stress adaptation processes in green plants.

## Materials and methods

### Amino acid sequence alignments and analysis

**Figure 1:**
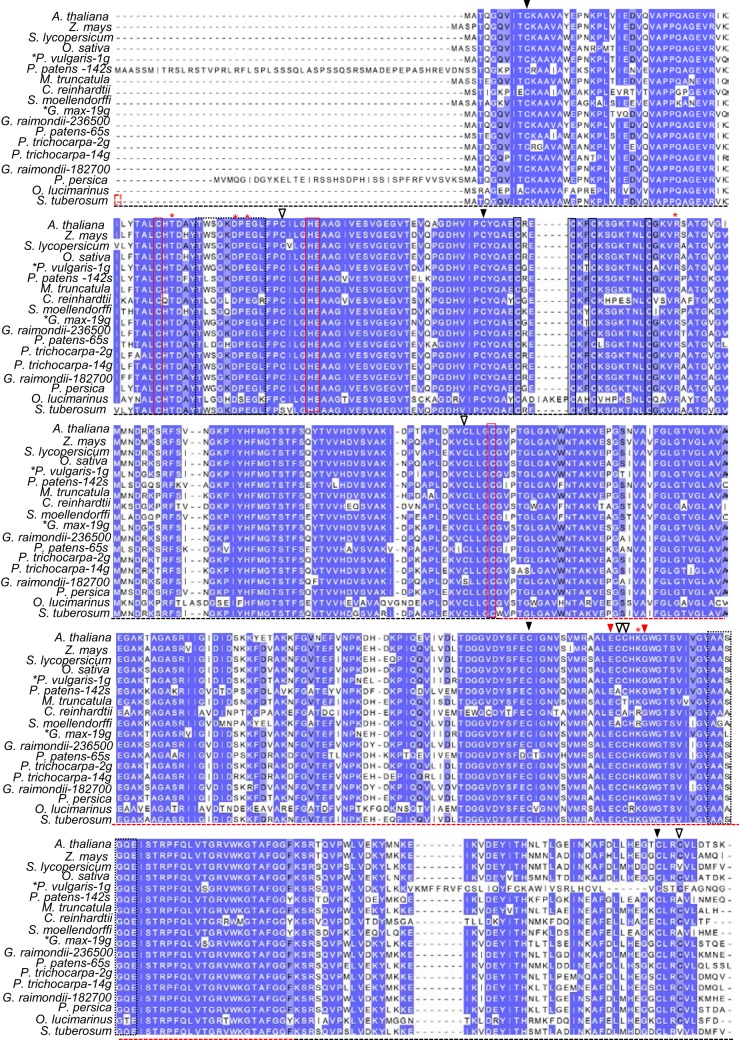
**Conserved features of plant GSNOR proteins.** Dark blue, light blue, and uncolored residues, respectively, refer to 100%, ≥75%, and <75% sequence conservation. Black and red dotted horizontal lines demarcate the catalytic and NADH-binding domains, respectively, as reported in crystal structures of tomato GSNOR (PDB code 4DL9). Residues coordinating structural and catalytic zinc atoms are outlined by solid black and red boxes, respectively. Red asterisks denote substrate-binding amino acids according to Kubienová et al. (2013). Dotted black boxes highlight flexible regions enclosing the active site. Red arrowheads (▼) indicate positions of the *hot5-1* and *hot5-3* missense mutations (Lee et al., 2008). Open (∇) and closed (▼) black arrowheads designate ex-zinc cysteines found in most and all plant sequences, respectively. *Z. mays*: maize. *S. lycopersicum*: tomato. *O. sativa*: rice. *P. vulgaris*: bean. *P. patens*: Physcomitrella. *M. truncatula*: Medicago. *C. reinhardtii*: Chlamydomonas. *S. moellendorffii*: Selaginella. *G. max*: soybean. *G. raimondii*: cotton. *P. trichocarpa*: poplar. *P. persica*: peach. *O. lucimarinus*: Ostreococcus. *S. tuberosum*: potato. *S. cerevisiae*: budding yeast. *H. sapiens*: human. Black asterisks: species with genomes predicted to encode additional paralogs, but only transcript-supported sequences are shown. Accession numbers can be found in Supp. Table 1 and an alignment of additional plant sequences not currently supported by transcript data is presented in Supp. Figure 1.

Predicted genes encoding GSNOR from green plants were retrieved from NCBI Genbank and Phytozome v9.1 (Goodstein et al., 2012) using Arabidopsis GSNOR (At5g43940, ADH2) as the tblastn query. *GSNOR* copy number was assessed with Phytozome and NCBI tblastn algorithms by querying predicted GSNOR- encoding genes against genomic reads from a particular plant species. Similar hits were considered duplicates if 5′ and 3′ intragenic and intron nucleotide sequences were >99% identical. Predicted GSNORs were also aligned via ClustalW (Larkin et al., 2007) with the Arabidopsis protein most similar to GSNOR (alcohol dehydrogenase, AtADH1, At1g77120), and sequences that cladded with AtADH1 were culled. Bacterial, metazoan, and fungal orthologs discussed in the text were uncovered through an NCBI tblastn search with *E. coli*, *Saccharomyces cerevisiae*, and human GSNOR queries, respectively. ClustalW sequence alignments were made with Jalview (Waterhouse et al., 2009). Deduced protein sequences were included in Figure 1 if transcript evidence (i.e., RNAseq and/or ESTs spanning the coding sequence) was available through Phytozome. Phylogenetic trees were drawn from aligned sequences in EvolView (Zhang et al., 2012). N-terminal targeting peptide searches were performed with Predotar (Small et al., 2004) and MITOPROT (Claros, 1995). Mitochondrial targeting peptides encoded by *GSNOR* 5′ intragenic regions were identified by six-frame translation of the first 500 base pairs upstream of the start codon using the intron splice rules for Arabidopsis (Hebsgaard et al., 1996).

### GSNOR structural assessments

Crystal structures of GSNOR from tomato (4DL9), human (1MP0), and Arabidopsis (4JJI and 4GL4) were obtained from the Protein Data Bank. Graphics were made with PyMOL v 1.6 (PyMOL).

### Plant material and growth conditions

The Arabidopsis GSNOR null mutants, *hot5-2* (also known as *atgsnor1-3*) (Col-0 background) and *hot5-4* (WS background), have been described previously (Lee et al., 2008). Unless otherwise indicated, plants were grown in soil in growth chambers on 16 h days (150 μMol m^−2^ s^−1^ light intensity) and a 21/19°C day/night temperature cycle. For analysis of the number of branches produced by wild-type and mutant plants, plants were grown as above and their height and branch pattern and numbers were measured 8 weeks after germination.

### Generation and visualization of GSNOR-GFP fusions in plants

Green fluorescent protein (GFP) was fused in frame 3′ to the Arabidopsis *GSNOR* genomic DNA (including 754 bp and 180 bp of 5′ and 3′ UTR, respectively) as follows. Genomic DNA was cloned into pENTR/D TOPO (Life Technologies). GFP was obtained from pMDC83 (Curtis and Grossniklaus, 2003). *Hind*III and *Xba*I sites were added to GFP and GSNOR-encoding sequences with primers GGATGCAAGCTTAGTAAAGGAGAAGAAC and TCTCTAGATTATTTGTATAGTTCATCCATGC (GFP) and primers CGTTGTG TCCTCGATACCAGCAAGCTTGTCTCTAGATGACTATATGGGTCCTCTCTGC and GCAGAGAGGACCCATATAGTCATCTAGAGACAAGCTTGCTGGTATCGAGGAC ACAACG (GSNOR). PCR products were digested with *Hind*III and *Xba*I, purified, and ligated. The GSNOR-GFP clone was then subjected to PCR with primers GGTACCGAA TTCCTAGAGTACAACCTC and TCGAGTGCGGCCGCTAAACT ATATGATTAG to add *Eco*RI and *Not*I sites, respectively. The PCR product was then ligated into *Eco*RI/*Not*I-digested pENTR2B. Constructs were cloned into the binary vector pBIB Basta-GWR (Gou et al., 2010) with LR Clonase II (Invitrogen), after which the sequence was verified.

The GSNOR-GFP translational fusion was then transformed into *hot5-2* plants, and homozygous transformants were identified by Basta screening and western blots. Three independent transgenic lines were analyzed. Whole seedlings and roots were imaged 7 days after germination on minimal nutrient medium with 0.5% sucrose. Whole flowers and reproductive structures were analyzed using stage 13 or 14 flowers as indicated in the figure legend. Images were obtained using an Olympus Fluoview FV1000 confocal microscope, with the exception of the whole seed and isolated stamens, which were imaged using conventional fluorescence microscopy with a NIKON Eclipse E800 microscope equipped with a SPOT camera (Molecular Diagnostic).

### Microarray analysis

Wild-type (Col) and *hot5-2* Arabidopsis plants were grown on soil in a growth chamber on a 12 h light, 21°C/12 h dark, 19°C cycle for 25 days after germination. Four biological replicates of wild-type and *hot5-2* leaves were sampled for RNA extraction 1 h before the end of the light period. A two-color, dye swap hybridization was performed on a long-oligonucleotide array chip by the Galbraith lab (University of Arizona) according to published methods (Zanetti et al., 2005; Zhang et al., 2008). Data were analyzed with Robin Version 0.9.6 BETA (Lohse et al., 2010). Differentially-expressed genes were identified by two criteria: (1) change in expression greater than 2-fold, and (2) a *t*-test *p*-value <0.01. Normalization of expression data, analysis using a linear model, and Benjamini and Hochberg false discovery rate correction for multiple comparisons were performed using LIMMA (Smyth and Speed, 2003).

## Results

### Catalytic and zinc-coordinating residues are conserved in GSNOR from green plants

To identify potentially novel shared motifs in plant GSNORs, we employed the tblastn algorithms of Phytozome (Goodstein et al., 2012) and NCBI Gene Expression Omnibus (Altschul et al., 1997) to search for GSNOR sequences supported by transcriptional data. Eighteen unique, type III ADH-encoding cDNAs were obtained from a variety of monocots, dicots, mosses, and protists. Residues near the dimer interface of tomato GSNOR (Kubienová et al., 2013), whose mutation compromises thermotolerance in dark grown Arabidopsis seedlings (Lee et al., 2008), are notably found in all plant sequences, while a glycine that when mutated to aspartate diminishes GSNOR activity, but confers enhanced paraquat resistance (Chen et al., 2009), is present in all but two algae (Figure 1). Substrate- and NADH-enclosing clefts (Figure 1, black dotted boxes) are identical or contain conservative substitutions among moss and algal orthologs. Structural and catalytic zinc-coordinating residues (Figure 1, solid black and solid red boxes, respectively) and substrate-binding amino acids (red asterisks), as reported by Kubienová et al. (2013), are identical in all but two predicted proteins, which contain a single lysine to arginine, conservative substitution.

**Figure 2:**
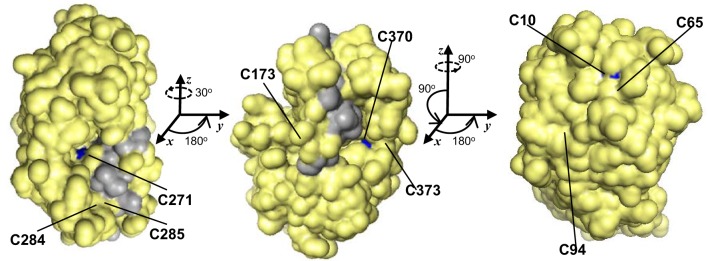
**Of nine positionally-conserved ex-zinc cysteines in GSNOR three are solvent-accessible.** Three orientations (rotation angles indicated) of a monomer of the Arabidopsis GSNOR dimer are shown with solvent accessible surface (PDB 4JJI, 1.8 Å res., R_free_ = 0.223) in yellow. Solvent-accessible ex-zinc cysteines are indicated in blue, and the dimer interface in gray. Images were made in PyMOL.

GSNOR is remarkably cysteine rich, with a mole percent cysteine of 3.84 % for the Arabidopsis protein, compared to the 1.37 % average for all proteins in the UniProtKB database (2013). Because cysteines can serve as key post-translational regulatory sites being modified by nitrosation, glutathionylation, or reversible oxidation, we analyzed the conservation of the nine extra non-zinc-coordinating cysteine residues (ex-zinc cysteines) in Arabidopsis GSNOR. Four are in all the transcript-supported plant sequences, two are substituted in one, two are substituted in two, and one differs in four organisms (Figure 1, solid and open black arrowheads), yielding an overall conservation of 93.8 % (i.e., on average, each cysteine is present in 17 of the 18 sequences). If additional plant genes are considered for which EST support is lacking, ex-zinc cysteine conservation is still 91.0% (Supp. Figure 1—see Supp. Table 1 for accession codes). Thus, the position and frequency of ex-zinc cysteines are highly conserved in plant GSNORs. We also examined the position of ex-zinc cysteines in the Arabidopsis GSNOR structure (PDB 4JJI, via (PyMOL) and found that three were solvent accessible (Cys-10, Cys-271, and Cys-370, Figures 2A–C), two of which—Cys-10 and Cys-271—are positionally conserved even in the human sequence. The structures of GSNOR from human (1MP0) and tomato (4DL9) showed similar solvent exposure of the homologous ex-zinc cysteines, suggesting these three residues may have conserved functions in regulating GSNOR activity.

### Most plant genomes encode one GSNOR protein predicted to be found in the cytosol

Encoded by a single gene, Arabidopsis GSNOR consists of nicotinamide cofactor-binding and catalytic domains and has two primary enzymatic activities—GSNO reductase and HMGSH dehydrogenase (Lee et al., 2008; Crotty, 2009; Kubienová et al., 2013). Interestingly, although *GSNOR* is found primarily as a single-copy gene in most other examined plant species (12 of 15 transcript-supported organisms, and 35 of 41 green plants analyzed), the dicots *Populus trichocarpa* (poplar) and *Gossypium raimondii* (diploid cotton) and the moss *Physcomitrella patens* (Physcomitrella) are predicted to have two *GSNOR*s. Although not yet supported by transcript data, gene families are also predicted in *Phaseolus vulgaris* (bean), *Glycine max* (soybean), and *Malus domestica* (apple; Supp. Figure 1). Moreover, in phylogenetic trees calculated by the average distance method with EvolView (Zhang et al., 2012), paralogs within the same species were more similar to one another than to orthologs in other species (data not shown). Thus, not only do most plant genomes encode one copy of GSNOR, but duplication of GSNOR-encoding genes has occurred recently and sporadically among plant species.

Extended N-termini, which could facilitate organelle targeting, are present in predicted GSNOR sequences from *Prunus persica* (peach) and Physcomitrella (Figure 1). Therefore, the Predotar (Small et al., 2004) program was used to search for putative endomembrane, plastid, and mitochondrial targeting peptides in all GSNORs. Of 50 input sequences, only one Physcomitrella paralog was predicted to have a mitochondrial targeting peptide. Analysis with MITOPROT (Claros, 1995) achieved similar results. Intriguingly, 5′ intragenic regions of GSNORs from Arabidopsis, rice, and *Selaginella moellendorfii* included cryptic splice sites that could give rise to putative mitochondrial targeting peptides, but intragenic regions from tomato, potato, Medicago, and *O. lucimarinus* did not. These results suggest GSNOR is a cytosolic enzyme in most plants.

We also noted predicted N- and C-terminal extensions in non-transcript-supported orthologs from two strains of *Micromonas pusilla* (an alga) and in apple that are too long to be signaling peptides (Supp. Figure 1). BLAST searches indicated *M. pusilla* extensions were formylglutathione hydrolase (FGH) domains, which catalyze the decomposition of S-formylglutathione (FGSH) to GSH and formate. FGHs are predicted in many green plants, albeit as separate gene products. FGSH is the product of the HMGSH dehydrogenase activity of GSNOR and is a key intermediate in formaldehyde detoxification (Staab et al., 2008). This observation suggests that, at least in the case of *M. pusilla* strains, NO and formaldehyde metabolism are intimately linked. The C-terminus of an apple GSNOR comprises a 4,5-DOPA dioxygenase (DOD), an enzyme in the biosynthesis of phenylpropanoid-derived betalain pigments that aid in plant defense (Georgiev et al., 2010), but transcriptional support has not been obtained for this fusion.

### Expression and localization of arabidopsis GSNOR

**Figure 3:**
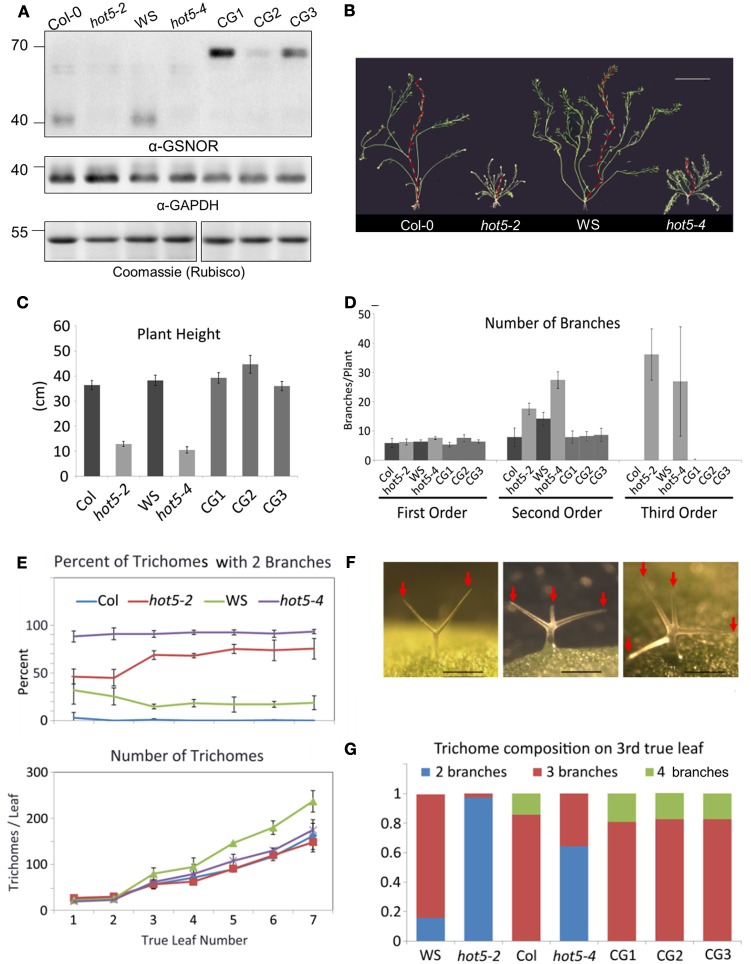
**Expression of GSNOR-GFP under its own promoter rescues multiple aspects of the *hot5-2* null phenotype. (A)** Immunoblots of leaf protein extracts probed with anti-Arabidopsis GSNOR antibodies (top) or anti-cytosolic GAPDH blots (middle). Rubisco large subunit (Coomassie stain, bottom). Plant genotypes are as indicated with CG1, 2, and 3 being independent homozygous T3 lines expressing GSNOR::GSNOR-GFP in the *hot5-2* background. **(B)** Shoot systems of indicated genetic backgrounds with primary inflorescence overlaid in red. Bar: 5 cm. **(C,D)** Shorter plant height **(C)** and inflorescence branching order **(D)** are rescued to wild-type levels in CG1, 2, and 3. **(E)** Trichomes with reduced branching are more numerous in *hot5-2* and *hot5-4* rosette leaves than in respective wild-type backgrounds. Error bars: *STD* **(F)** Left to right: trichomes from *hot5-2*, Col, Col. Bar: 0.2 mm. Red Arrows indicate trichome branches. **(G)** Trichome branching is rescued to wild-type levels in CG1, 2, and 3.

To determine the major sites of GSNOR function, we created a translational fusion of GPF at the C-terminus of GSNOR driven by native promoter sequence and transformed this construct into the *GSNOR* null mutant *hot5-2* (Lee et al., 2008). We carried forward three homozygous transgenic lines that express GSNOR-GFP (Figure 3A). Mutation of *GSNOR* results in reduced plant height and an increased number of inflorescences (Lee et al., 2008; Kwon et al., 2012). We quantified these differences in mature plants and found that while *hot5-2 and hot5-4* null mutants and corresponding wild-types (Col-0 and WS, respectively) produce a similar number of first-order inflorescence stems (arising from the rosette), mutants produce two-fold more second-order branches and often produce third-order branches, which are not observed in wild-type plants (Figures 3B–D). All three complemented lines showed quantitative restoration of wild-type height and branching patterns (Figures 3C,D), indicating that the GFP fusion did not compromise GSNOR function. We further observed reduced trichome branching in *hot5* null plants (Figures 3E,F), a previously unreported phenotype. Mutants primarily produced trichomes with only two branches, rather than the three or four typical of wild type (Figure 3F). 70% of *hot5-2* trichomes were two-branched compared to virtually zero in Col, and similar differences were detected between WS and *hot5-4* (Figure 3G). The absolute number of trichomes was marginally higher in WS leaves compared with those of *hot5-4*, in agreement with Holzmeister et al. (2011), while trichome abundance did not differ between Col and *hot5-2* (Figure 3E, lower panel), indicating the difference in trichome number must be due to a modifying gene present only in the WS background. Quantitation of trichome branching in the complemented lines showed that the GSNOR-GFP fusion protein restored branching to wild-type levels (Figures 3F,G).

**Figure 4:**
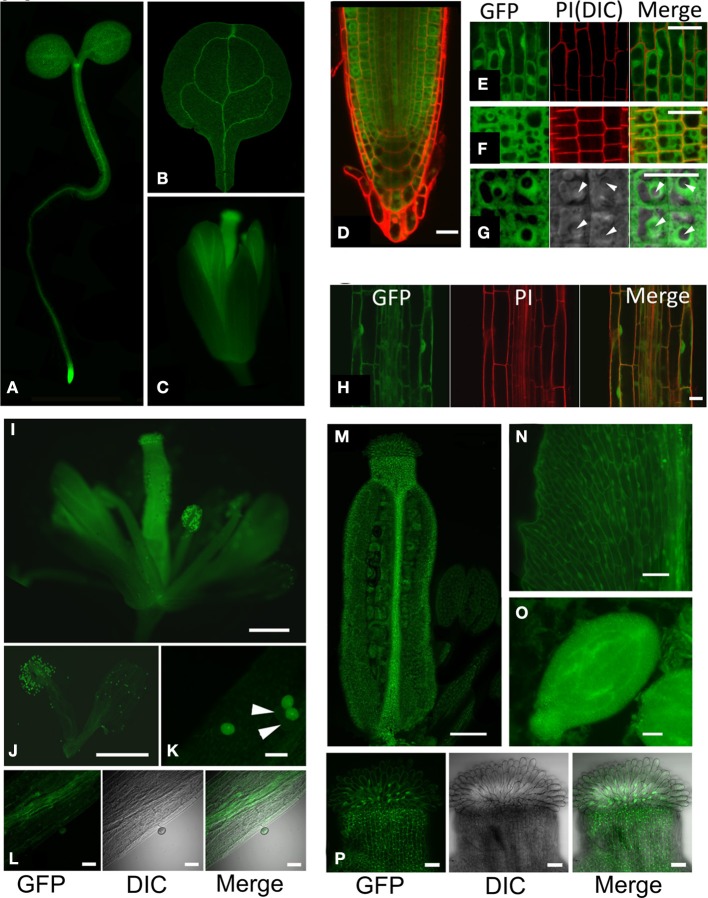
**GSNOR is expressed in various organs and developmental stages in Arabidopsis.** Localization of GSNOR was observed in plants transformed with GSNOR::GSNOR-GFP in the *hot5-2* background. All plants were homozygous for the GSNOR::GSNOR-GFP transgene, with the exception of those used for images in **(I–K)**. **(A)** Whole seedling. **(B)** Cotyledon. **(C)** Flower, stage 13. **(D)** GSNOR-GFP distribution and localization in root tip cells in optical cross section through the middle of the root. **(E)** GSNOR-GFP localization in root epidermal cells. **(F, G)** Optical cross section of GSNOR-GFP localization in root cortex cells at two different magnifications. Arrowheads: nucleolus. PI, Propidium Iodide staining; DIC, Differential Interference Contrast microscopy. **(H)** GSNOR-GFP localization in the root elongation zone. Bar: 20 μm **(D–H)**. **(I–P)** Localization of GSNOR-GFP in stage 14 flowers **(I)** stamens and petals **(J)** pollen **(K)** anther filaments **(L)** ovary **(M)** petals **(N)** seed at bending cotyledon embryonic stage **(O)** and stigma **(P)**. Arrowheads in **(K)** denote *hot5-2* pollen that do not express GSNOR-GFP due to segregation of the transgene in the heterozygote. Bar: 500 μm **(I,J)** 200 μm **(M)**, or 40 μm **(K,L, N–P)**.

Based on complementation of *hot5* phenotypes, the GFP fusion seemed suitable to assess GSNOR localization. Whole seedlings at the cotyledon stage and flower stage 13 were imaged to observe the tissue and subcellular localization of GSNOR-GFP (Figure 4). Fluorescence was observed throughout seedling and floral structures (Figures 4A–C), which correlates with gene expression data in public databases (Toufighi et al., 2005). The seedling apical meristem and root tip exhibited notably intense fluorescence (Figures 4A,D), while cotyledon, hypocotyl, root, and petal vascular tissue signals were highest (Figures 4A–C). Detailed observation of sections through the root tip (Figures 4E–G) show distribution in all root cell types with diffuse cytosolic and nuclear localization, but dramatic exclusion from the nucleolus. This is also evident in the elongating zone of the root, although nucleoli are considerably smaller in these cells (Figure 4H). GSNOR-GFP could also be detected in anther filaments, ovary, stigma, and petals (Figures 4I,J,M,N,P) of stage 14 flowers and was particularly enriched in pollen and seed (Figures 4K,O).

### Multiple pathogen response genes are downregulated in *hot5-2*

To assess global changes in the transcriptome due to GSNOR absence, we performed microarray analysis on 4-week leaves of *hot5-2* plants grown on a 12 h light cycle since the leaves of Col and *hot5-2* plants are most morphologically comparable at this age (Lee et al., 2008). Imposing stringent criteria of ≥2-fold changes with *p* ≤ 0.01 (FDR-corrected), we found 99 and 170 transcripts up- and downregulated, respectively, in *hot5-2* compared to Col-0. A complete list of transcripts is provided in Supp. Table 2 categorized by pathway as curated in MAPMAN (Usadel et al., 2009). This list was used to test for enriched categories of regulated genes (Usadel et al., 2006). One group of enriched genes was the “Stress Response” category (Table 1). Of 19 genes in this category, 13 were downregulated “biotic stress” genes, including pathogenesis-related protein 1 (PR1, ~6.9-fold), consistent with data from Feechan et al. (2005), four potential pathogen receptors (At1g59218, At3g04210, At3g50470, At3g11010), PR5, and other predicted herbivore and pathogen defense proteins. Of two upregulated “Stress” category genes, the defensin protein PDF1.2 was also reported to be upregulated in *GSNOR* antisense plants (Espunya et al., 2012).

**Table 1:** **Changes in “Stress Responsive” Transcripts in *hot5-2* vs. wild-type**.

**Gene**	**Protein**	**Stress**	**Fold-change (log2)**	***p*-value**
**UPREGULATED**					
At3g04720	PR4 (PATHOGENESIS-RELATED GENE 4)	Biotic	1.397	0.001	
At5g44420	PDF1.2; PDF1.2A; LCR77; Defensin	Biotic	1.017	0.001	
**DOWNREGULATED**					
At2g14610	PR1 (PATOGENESIS-RELATED GENE 1)	Biotic	−2.78	0	
At4g19820	Glycosyl hydrolase family 18 protein	Biotic	−2.25	0	
At2g37570	SLT1 (SODIUM AND LITHIUM TOLERANT 1)	Abiotic	−2.005	0.001	
At1g73330	DR4 (DROUGHT REPRESSED 4); peptidase inhibitor	Biotic	−1.882	0	
At3g50480	HR4 (HOMOLOG OF RPW8 4)	Biotic	−1.861	0.001	
At1g75040	PR5 (PATHOGENESIS-RELATED GENE 5)	Biotic	−1.821	0.001	
At3g11010	AtRLP34 (Receptor-like Protein 34)	Biotic	−1.8	0.002	
At1g24020	MLP423 (MajorLatexPprotein-LIKE PROTEIN 423)	Abiotic	−1.65	0	
At3g50470	HR3 (HOMOLOG OF RPW8 3)	Biotic	−1.502	0	
At2g43510	ATTI1; serine endopeptidase inhibitor	Biotic	−1.414	0	
At3g04210	Disease resistance protein (TIR-NBS class)	Biotic	−1.391	0.001	
At2g43530	Trypsin inhibitor Defensin-like protein	Biotic	−1.282	0.001	
At1g59218	Disease resistance protein (CC-NBS-LRR class)	Biotic	−1.272	0.001	
At3g48080	Lipase class 3 family protein	Biotic	−1.238	0.001	
At1g72260	THI2.1 (THIONIN 2.1)	Biotic	−1.117	0.001	
At2g03720	MRH6 (morphogenesis of root hair 6)	Abiotic	−1.083	0.008	
At1g19670	COR1 (CORONATINE-INDUCED PROTEIN 1); CHL1 (CHLOROPHYLLASE 1)	Abiotic	−1.013	0.001	

p ≤ 10^−*8*^ listed as = 0.

These results are consistent with the involvement of NO in pathogen responses, and prompted us to employ the Arabidopsis eFP browser (Toufighi et al., 2005) and TAIR gene annotations to determine if other pathogen response-linked genes, including those in the “Not Assigned” category (the largest category with 76 genes) were also differentially regulated in *hot5-2*. The eFP data include response to the oomycete *Phytophthora infestans*, the bacterium *Pseudomonas syringae* (virulent and avirulent), the fungi *Botrytis cinerea* and *Erysiphe orontii*, as well as induction by non-host bacteria and several elicitors. As highlighted in Supp. Table 2, 43 additional genes that can be linked to pathogen response are down-regulated (e.g., ACD6, At4g14400; DIR1, At5g48485), making a total of 56 of 170 downregulated genes linked to pathogen response. In contrast, only 13 additional upregulated genes can be linked to pathogen response. Since ~12% of Arabidopsis genes are pathogen defense-related (The Arabidopsis Initiative, 2000), we conclude the absence of *GSNOR* disproportionately downregulates pathogen response genes.

### An unusual class of glutaredoxins are upregulated in *hot5-2*

Given the significant interplay between GSNO levels and GSH-controlled redox homeostasis (Staab et al., 2008), it was of interest that genes in the “Redox” category were also enriched. Intriguingly, six cytosolic ROXY-class glutaredoxins (GRXs) and an atypical chloroplast-localized thioredoxin (TRX) were upregulated in *hot5-2* (Table 2 and Supp. Table 2). GRXs and TRXs are small oxidoreductases that regulate the thiol redox state of other proteins (Meyer et al., 2012). Four of the six up-regulated GRX genes are closely linked on chromosome 4 and share 91–95% identity, while two others on chromosomes 5 and 1 are 72–75% and 58–60% identical to the chromosome 4 genes, respectively, and 59% identical to each other. All six GRXs belong to a plant-specific GRX family containing a monocysteine active site. Thus, these GRX proteins likely act as monothiol GRXs (Herrero and De La Torre-Ruiz, 2007). One cytosolic, monothiol ROXY GRX (ROXY20), which is divergent from the upregulated genes (~40% identical), is downregulated in *hot5-2*. Examination of publically available expression patterns of these regulated ROXY genes (Schmid et al., 2005) indicates that during normal plant growth the *hot5-2* upregulated *ROXY* genes have low transcript levels in stems, senescing leaves, and the shoot apex, while *ROXY20* shows the opposite pattern. Thus, these GRX proteins are likely to serve different functions. Currently the redox targets of these cytosolic GRXs and the unusual chloroplast TRX are unknown, but increased expression of the corresponding genes indicates that mutation of *GSNOR* alters redox homeostasis and potentially the targets of these oxidoreductases.

**Table 2:** **Changes in “Redox” Transcripts in *hot5-2* vs. wild-type**.

**Gene**	**Protein**	**Function/Localization**	**Fold-change (log2)**	***p*-value**
**UPREGULATED**				
At4g15690	Glutaredoxin S5	Glutaredoxin; cytosolic; CxxS active site	2.788	6.94 E-05
At4g15670	Glutaredoxin S7	Glutaredoxin; cytosolic; CxxS active site	2.758	8.85 E-05
At4g15660	Glutaredoxin S8	Glutaredoxin; cytosolic; CxxS active site	2.088	0
At4g15700	Glutaredoxin S3	Glutaredoxin; cytosolic; CxxS active site	1.786	0
At1g03020	Glutaredoxin S1	Glutaredoxin; cytosolic; CxxS active site	1.595	0
At5g04720	ACHT5 (ATYPICAL CYS HIS RICH THIOREDOXIN 5)	Thioredoxin; chloroplast; CGGC active site	1.128	0.001
At5g18600	Glutaredoxin S2	Glutaredoxin; cytosolic; CxxS active site	1.064	0.001
**DOWNREGULATED**				
At5g11930	Glutaredoxin C10	Glutaredoxin; cytosolic; CxxS active site	−1.155	0.001
At1g20620	CAT3	Catalase; peroxisome; cytosol?	−1.108	0.002

p ≤ 10^−*8*^ listed as = 0.

### Changes in *hot5-2* gene expression indicate other processes impacted by NO homeostasis

Another enriched category of genes that presents a consistent picture of changes in the GSNOR mutant is the “Signaling” category, in which 14 of 15 genes are down regulated and half of the downregulated genes are involved in calcium signaling, including multiple calmodulins, calmodulin-like proteins and calreticulins (Supp. Table 2). The single upregulated gene in this category is ATCP1 (CALCIUM BINDING PROTEIN 1, At5g49480), which also has sequence similarity to calmodulin. These data provide a direct link of GSNO to calcium signaling.

Although “Transcription” was not a specifically enriched category, consideration of regulated transcription factors (Supp. Table 1) shows that three of the most highly upregulated genes encode BHLH proteins involved in iron deficiency responses (BHLH100, At2g41240; BHLH039, At3g5698; BHLH038, At3g56980) (Wang et al., 2013). The potential targets of these transcription factors in leaves are unknown, although in roots they can activate genes required for iron uptake. Among downregulated genes in the transcription category, it is notable that there are three AP2/EREBP transcription factors, which are involved in ethylene responses, including the most strongly downregulated gene in this category, TINY (At5g11590; >5-fold decreased) (Sun et al., 2008).

## Discussion

The pleiotropy of Arabidopsis *GSNOR* loss-of-function mutants indicates that enzymatic control of GSNO levels is essential for competitive viability (Lee et al., 2008; Kwon et al., 2012) (Feechan et al., 2005). *In vitro*, GSNOR catabolizes GSNO and HMGSH, metabolites generated from NO- and formaldehyde, respectively. Because NO can lead to the generation of reactive nitrogen species (RNS) and formaldehyde can produce ROS (Staab et al., 2008; Kubienová et al., 2013), GSNOR is likely critical to regulation of downstream physiological and pathological effects of RNS and ROS in plants. The evolutionary conservation of GSNOR in eukaryotes and bacteria, which was recognized over a decade ago (Liu et al., 2001), further indicates this enzyme has similar functions in multiple domains of life. Here we have identified conserved features of *GSNOR* genes and their encoded proteins in plants, determined aspects of tissue and cellular localization of GSNOR, and shown that its absence impacts expression of pathogen response, redox, and calcium signaling genes in Arabidopsis. While this work is primarily descriptive, it offers insight into the molecular consequences of GSNOR loss and therefore can serve as a prospectus for future mechanistic studies.

*GSNOR* was found as a single-copy gene in 35 of 41 plant species, with seven instances of recent duplication events (data not shown). De Smet et al. (2013) theorize that the preponderance of single copy genes in organisms that have undergone whole genome duplication (such as Arabidopsis) is not random, but rather indicates that these genes impair fitness when present in multiple copies, possibly due to overly-robust activity of their encoded proteins. In any event, GSNOR copy number appears to be under strong selective pressure.

All putative eukaryotic GSNORs we examined show high conservation and are unusually rich in cysteines, which are highly positionally conserved among plant orthologs (Figures 1, 2 and Supp. Figure 1). Common functions of protein cysteine residues include coordination of metal atoms (i.e., copper and zinc), covalent catalysis, extracellular adhesion, and redox sensing (Wang et al., 2012). Most of the ex-zinc cysteines in Arabidopsis GSNOR were found to be inaccessible to solvent (Figure 2; Crotty, 2009), suggesting their primary function may be structural. Intriguingly, however, three cysteines that are positionally conserved between plants and animals are solvent accessible (Figure 2, blue patches). These residues may serve as sites of post-translational regulation via, for instance, glutathionylation or S-nitrosation. Residues that bind HMGSH or coordinate zinc (Kubienová et al., 2013) were found in all predicted plant proteins (Figure 1 and Supp. Figure 1), indicating GSNO reductase and HMGSH dehydrogenase activities are probably general features of plant GSNORs. GSNORs from Arabidopsis and tomato exhibit ~10-fold higher velocity of NADH-dependent GSNO reduction than NAD^+^-dependent HMGSH oxidation to formylglutathione (Crotty, 2009; Kubienová et al., 2013), but the high ratio of NAD^+^ to NADH in most living cells would favor the dehydrogenase reaction. This could be circumvented by cofactor recycling, wherein NADH produced from HMGSH oxidation is employed for GSNO reduction (Staab et al., 2008). Thus, GSNO catabolism and formaldehyde detoxification may be partially interdependent processes. This scenario is not unreasonable, as ROS and RNS are known to contribute to the formation of one another (Molassiotis and Fotopoulos, 2011). The mitochondrion is a major site of ROS production in eukaryotes. GSH and NO concentrations are reported to be high in Arabidopsis mitochondria (Wang et al., 2010; Koffler et al., 2013), and mitochondrial enzymes have been identified as nitrosation targets (Millar and Day, 1996; Palmieri et al., 2010). There is no transcriptional evidence for mitochondrion-localized GSNORs apart from one Physcomitrella homolog (Figure 1), but the existence of cryptic target peptide-encoding sequences in frame with GSNORs from diverse taxa (data not shown) underscores possible mitochondrial localization in a common evolutionary ancestor.

Visualization of GSNOR-GFP provided clear evidence for cytosolic and nuclear localization of this protein throughout the plant, with potentially higher concentrations in vascular tissues and very noticeable exclusion from the nucleolus. Though GSNOR lacks a nuclear localization signal, it may be transported in association with another protein. We saw no evidence of GSNOR-GFP in mitochondria, but the upstream region with potential to encode a mitochondrial targeting peptide was not included in our construct. Our results of organ and tissue localization do not fully agree with a previous report from Espunya et al. (2006); while they found high levels of activity in roots, no activity was detected in the hypocotyl or cotyledons, which show significant fluorescence in our study. Consistent with our observations, their immunocytochemistry showed GSNOR in all cell types of the root meristematic zone. However, the inner cortex of the root elongation zone appeared to lack protein when localized by immunocytochemistry, in contrast to our observations. Overall, GSNOR appears to function in essentially all plant cell types. These protein data are augmented by publically available transcript analysis in Arabidopsis, which show virtually ubiquitous expression of GSNOR mRNA, further supporting the role of this enzyme in multiple plant processes.

A well-documented feature of GSNOR null Arabidopsis is a multi-branching phenotype (Lee et al., 2008), which has been suggested to arise from impact on auxin transport and function, and/or cytokinin signaling (Kwon et al., 2012). Replacement of wild-type GSNOR with GSNOR C-terminally fused to GFP produced plants with quantitatively normal branching patterns, indicating GSNOR-GFP effectively functions like the wild-type protein in processes required for normal branching. The GSNOR-GFP transgene also rescued reduced trichome branching, a previously unrecognized phenotype of the null mutant. Arabidopsis trichomes are single cells, so reduced branching results from alteration of cell morphogenesis, which involves a wide range of basic processes (Hülskamp, 2000). How absence of GSNOR reduces trichome branches is not known, but notably, Arabidopsis trichomes have two to four times the concentration of GSH compared to other epidermal cells (Gutiérrez-Alcalá et al., 2000). High GSH may increase the deleterious effects of the absence of GSNOR specifically in these cells. GSNOR-GPF also rescued this phenotype, implying a role for NO homeostasis in basic cell morphological processes. The pleiotropy of the *GSNOR* loss-of-function phenotype suggests enhanced nitrosation of one or more proteins interferes with their normal physiological functions. However, there are only a few known *in vivo* targets of nitrosation in plants: the Arabidopsis cytokinin signal relay kinase AHP1 is negatively regulated via nitrosation of a conserved cysteine (Feng et al., 2013), while nitrosation of the F box protein TIR1 has been proposed to modulate auxin signaling (Terrile et al., 2012). Thus, the strong morphological phenotypes of *hot5-2* and *hot5-4* plants may reflect uncoordinated cytokinin and auxin crosstalk caused by aberrant nitrosation of TIR1 and AHP1. Observed changes in pathogen sensitivity may have a similar etiology. Indeed, salicylic acid (SA) signaling has been shown to be downreguated by GSNO-mediated nitrosation of NPR1 (Tada et al., 2008). Further work with the GSNOR null mutant will be required to determine any alterations in nitrosated or glutathionylated targets.

Comparison of transcript levels between a *GSNOR* null mutant and wild type Arabidopsis on microarrays revealed an enrichment in transcripts involved in pathogen responses, redox regulation, and calcium signaling, as well as upregulation of transcription factors involved in iron responses and downregulation of ethylene responsive transcription factors. The extensive downregulation of pathogen defense related and responsive genes supports the observations of Feechan et al. (2005), who reported the null mutant had reduced R gene mediated, basal and non-host resistance to pathogens. While the Arabidopsis homolog of human CR1 was not transcriptionally higher in the *hot5-2* mutant, as might be expected if it provided compensatory activity, *GRX*s and a *TRX* were notably upregulated. TRXs and GRXs can mediate denitrosation and deglutathionylation, respectively (Benhar et al., 2009; Zaffagnini et al., 2013), such that they could function to reverse an increase in these modifications due to excess GSNO in the mutant. Although the contribution of TRXs and GRXs to the acclimation of GSNOR-deficient plants to nitrosative stress has not been assessed, these microarray data strongly implicate the involvement of GRXs in plant SNO homeostasis. The microarray data also support proposed linkages between NO and calcium signaling (Courtois et al., 2008), as five calmodulins or calcium binding proteins are downregulated and one is upregulated in *hot5-2*.

We also compared the results of our microarrays with previous studies aimed at identifying NO regulated genes that used NO donors or NO synthase inhibitors to induce changes in gene expression in Arabidopsis (Parani et al., 2004; Besson-Bard et al., 2009). We found very limited overlap with potentially NO-regulated transcripts. For genes induced by application of 0.1 or 1.0 mM SNP (Parani et al., 2004), only four genes were also upregulated in *hot5-2*: the calmodulin-like ATCP1 (At5g49480), WRKY40 (At1g80840), a UDP glucosyl transferase (At1g05560) and a 67 amino acid unknown (At4g27654). Compared to transcripts regulated by a NOS inhibitor, in which downregulated genes were proposed to be normal targets of NO, again only four transcripts behaved similarly in our experiments (DIN10, At5g20250; XTR8,At3g44990; LTP1,At2g45780; unknown, At3g56360). We may see little overlap with these prior investigations because they examined short-term manipulation of NO levels, whereas GSNO and NO are chronically deregulated in *hot5-2*. In any event, changes in gene expression in the absence of GSNOR reflect a long-term metabolic adjustment required to cope with excess GSNO and the pathways it normally regulates.

GSNOR clearly plays a role in biotic stress adaptation, but how a GSNOR-DOD fusion such as that identified in a homolog from apple (Supp. Figure 1) would function *in vivo* is not clear, and this may be an artifact of early phase sequencing. Using the Toronto Bio-Analytic Resource (BAR) Arabidopsis gene expression data compendium (Toufighi et al., 2005), it was revealed that *GSNOR* transcription strongly correlated with NINJA [*r*^2^ = 0.62, a jasmonic acid (JA) response corepressor] and PMR5 (*r*^2^ = 0.59, a protein whose absence affords greater resistance to biotrophic fungi that cause powdery mildew). Both PMR5 and NINJA negatively regulate pathogen-induced defense signaling—PMR5 contributes to JA-independent fungal disease susceptibility (Vogel et al., 2004), while NINJA activity is curtailed following JA-induced, COI1-dependent proteasomal degradation of JAZ repressor proteins (Pauwels et al., 2010; Sheard et al., 2010). While the association of these pathogen response genes with *GSNOR* is only correlative, it can be inferred that GSNOR might also work to dampen biotic stress responses in the absence of elicitation. This would explain why *GSNOR* over-expression and RNAi-mediated knockdown served to respectively diminish and enhance systemic acquired tolerance and basal tolerance to a *P. syringae* and *Peronospora parasitica* (Rustérucci et al., 2007). This also harmonizes well with the observation that GSNOR positively affects SA signaling (Feechan et al., 2005), since JA and SA operate antagonistically to one another.

In summary, GSNOR appears to be a ubiquitously-expressed, cytosol-localized protein that regulates shoot morphology, pathogen defense responses, and NO homeostasis. Aberrant nitrosation of auxin, cytokinin, and SA response regulator proteins, among others, likely contribute to aspects of *hot5* null mutant phenotypes. Diminished branching in GSH-rich trichome cells further underscores the role of GSNOR in maintaining the cellular reduction potential, and its conserved, solvent-accessible cysteines may function as NO sinks or serve a regulatory role. The upregulation of transcripts of a class of GRXs is a particularly promising discovery, as some GRXs and TRXs catalyze deglutathionylation and denitrosation. Understanding how GRXs may compensate for loss of GSNOR and how GSNOR activity may be regulated through its conserved, solvent accessible cysteine residues will help to clarify the role of GSNOR in plant biology.

## Conflict of interest statement

The authors declare that the research was conducted in the absence of any commercial or financial relationships that could be construed as a potential conflict of interest.
