# Supplementary material for: S-nitrosoglutathione reductases are low-copy number, cysteine-rich proteins in plants that control multiple developmental and defense responses in Arabidopsis
Source: Front Plant Sci. 2013 Nov 5;4:430. doi: 10.3389/fpls.2013.00430 (PMC3817919; doi:10.3389/fpls.2013.00430)
Supplement: Supplementary file 1 [file Presentation1.PPTX]

## Slide 1
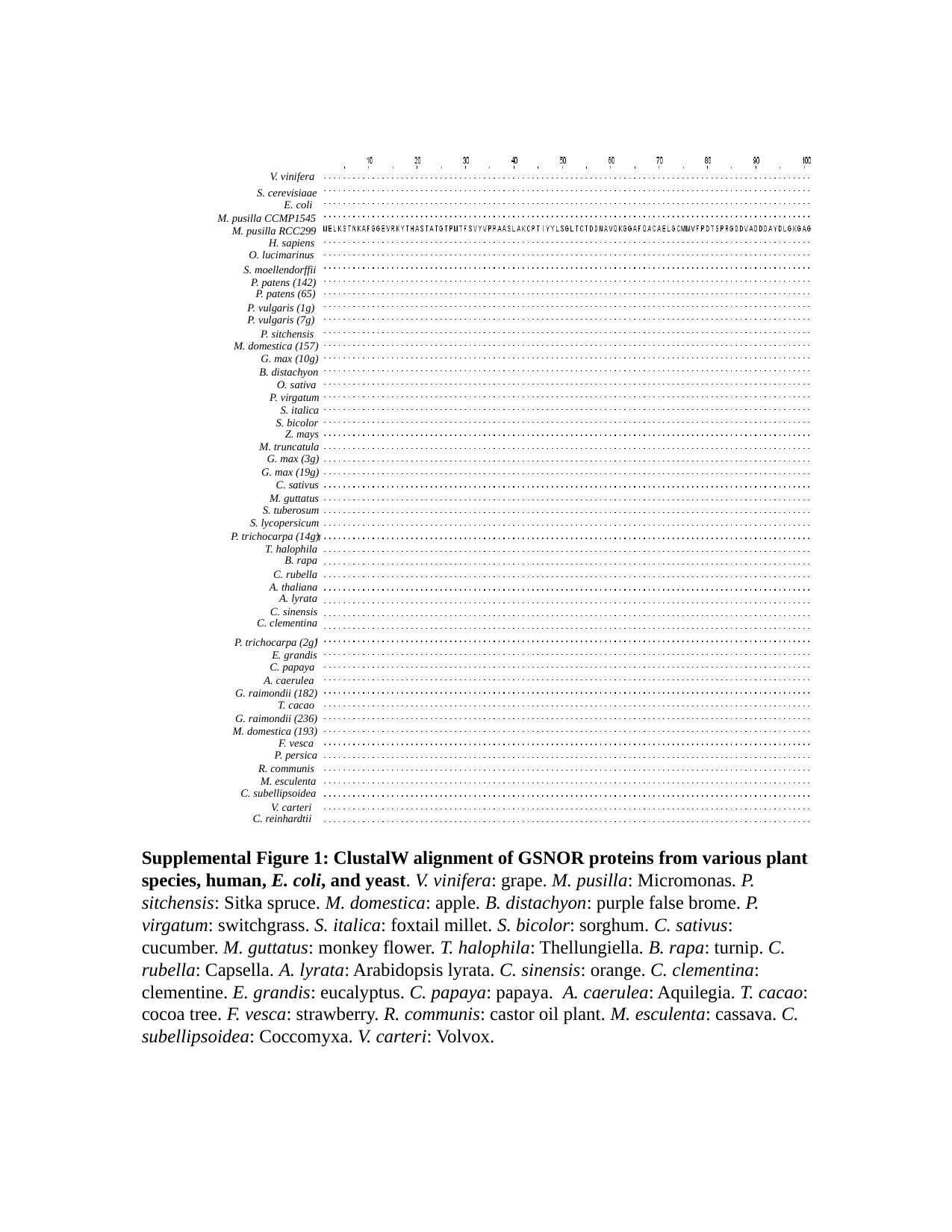

V. vinifera
S. cerevisiaae
E. coli
M. pusilla CCMP1545
M. pusilla RCC299
H. sapiens
O. lucimarinus
S. moellendorffii
P. patens (142)
P. patens (65)
P. vulgaris (1g)
P. vulgaris (7g)
P. sitchensis
M. domestica (157)
G. max (10g)
B. distachyon
O. sativa
P. virgatum
S. italica
S. bicolor
Z. mays
M. truncatula
G. max (3g)
G. max (19g)
C. sativus
M. guttatus
S. tuberosum
S. lycopersicum
P. trichocarpa (14g)
T. halophila
B. rapa
C. rubella
A. thaliana
A. lyrata
C. sinensis
C. clementina
P. trichocarpa (2g)
E. grandis
C. papaya
A. caerulea
G. raimondii (182)
T. cacao
G. raimondii (236)
M. domestica (193)
F. vesca
P. persica
R. communis
M. esculenta
C. subellipsoidea
V. carteri
C. reinhardtii
Supplemental Figure 1: ClustalW alignment of GSNOR proteins from various plant species, human, E. coli, and yeast. V. vinifera: grape. M. pusilla: Micromonas. P. sitchensis: Sitka spruce. M. domestica: apple. B. distachyon: purple false brome. P. virgatum: switchgrass. S. italica: foxtail millet. S. bicolor: sorghum. C. sativus: cucumber. M. guttatus: monkey flower. T. halophila: Thellungiella. B. rapa: turnip. C. rubella: Capsella. A. lyrata: Arabidopsis lyrata. C. sinensis: orange. C. clementina: clementine. E. grandis: eucalyptus. C. papaya: papaya. A. caerulea: Aquilegia. T. cacao: cocoa tree. F. vesca: strawberry. R. communis: castor oil plant. M. esculenta: cassava. C. subellipsoidea: Coccomyxa. V. carteri: Volvox.

## Slide 2
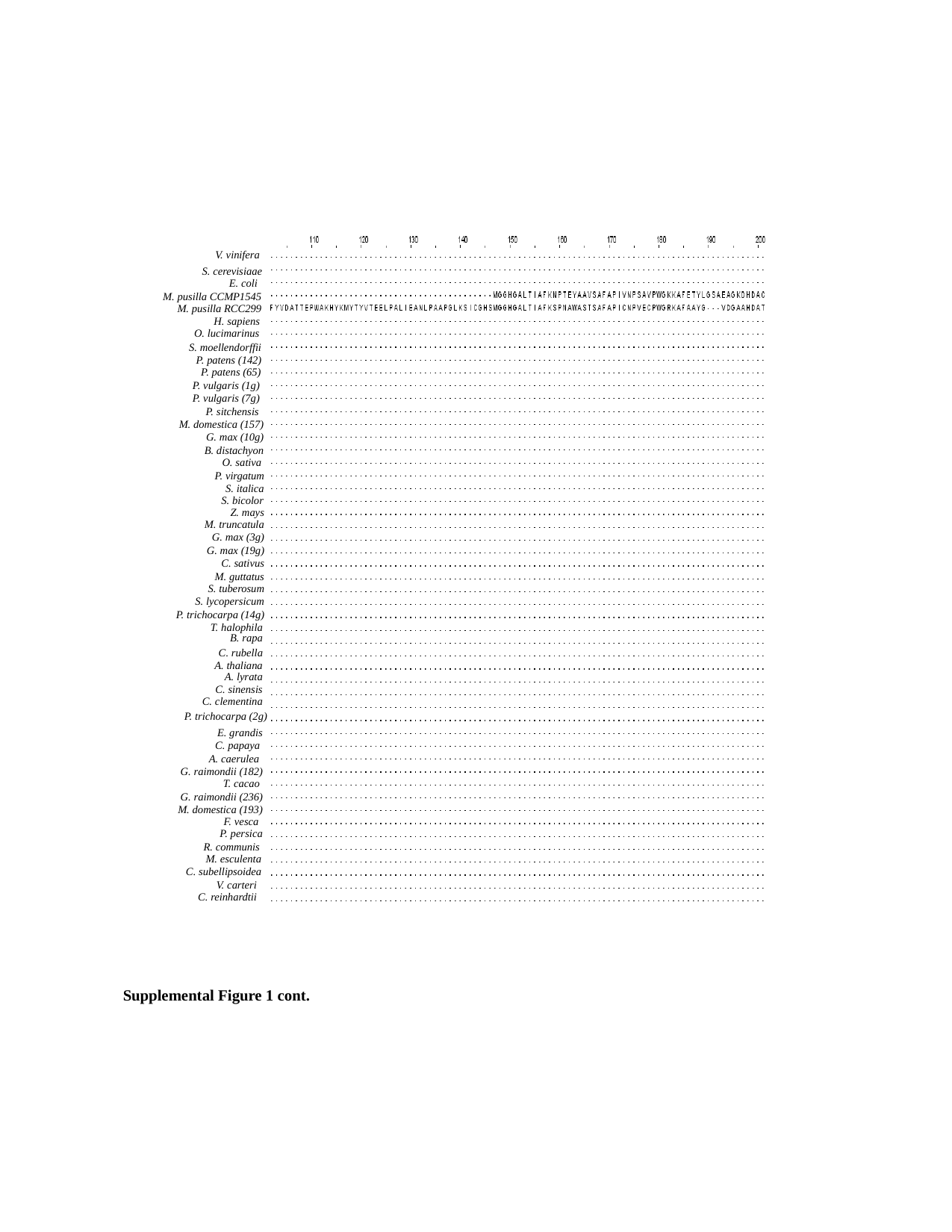

V. vinifera
S. cerevisiaae
E. coli
M. pusilla CCMP1545
M. pusilla RCC299
H. sapiens
O. lucimarinus
S. moellendorffii
P. patens (142)
P. patens (65)
P. vulgaris (1g)
P. vulgaris (7g)
P. sitchensis
M. domestica (157)
G. max (10g)
B. distachyon
O. sativa
P. virgatum
S. italica
S. bicolor
Z. mays
M. truncatula
G. max (3g)
G. max (19g)
C. sativus
M. guttatus
S. tuberosum
S. lycopersicum
P. trichocarpa (14g)
T. halophila
B. rapa
C. rubella
A. thaliana
A. lyrata
C. sinensis
C. clementina
P. trichocarpa (2g)
E. grandis
C. papaya
A. caerulea
G. raimondii (182)
T. cacao
G. raimondii (236)
M. domestica (193)
F. vesca
P. persica
R. communis
M. esculenta
C. subellipsoidea
V. carteri
C. reinhardtii
Supplemental Figure 1 cont.

## Slide 3
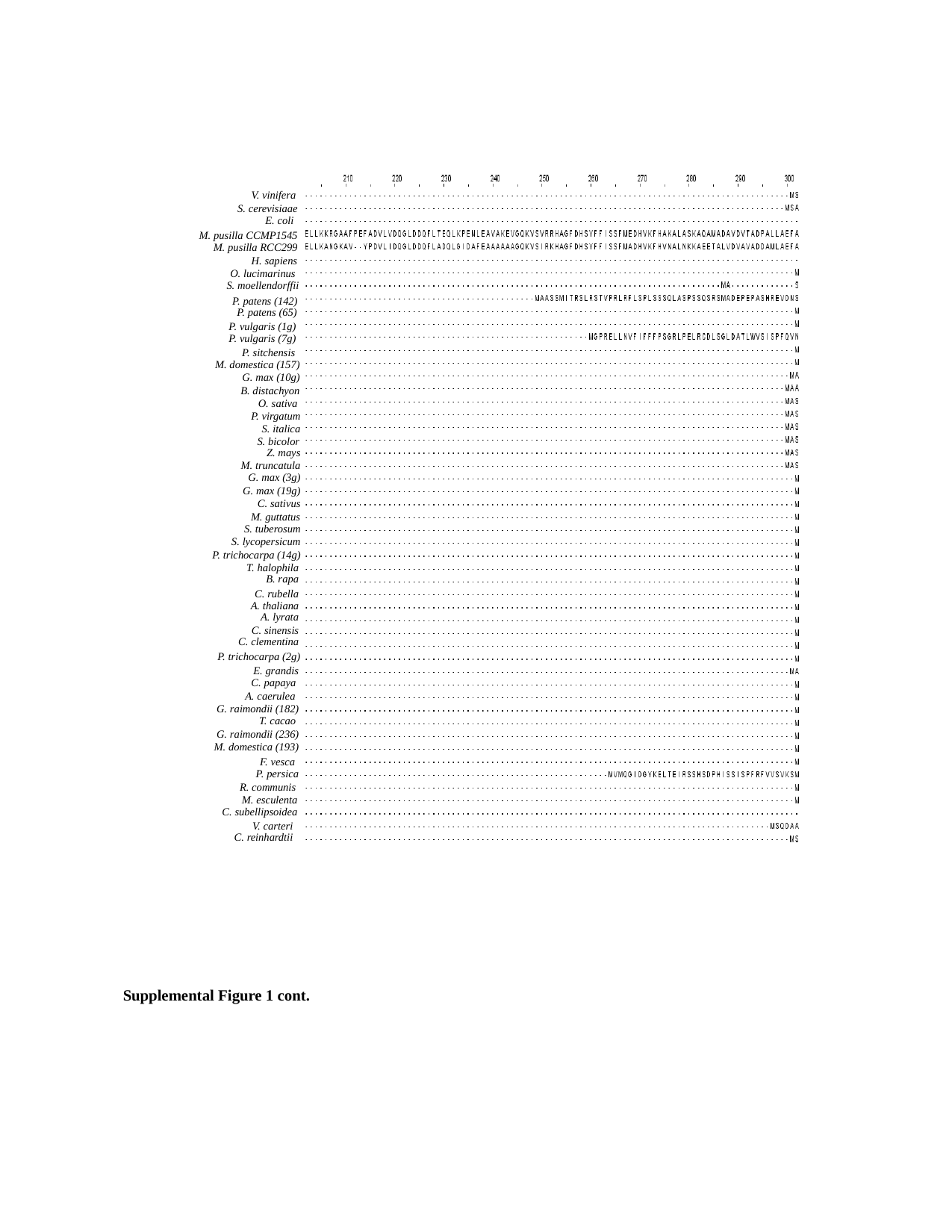

V. vinifera
S. cerevisiaae
E. coli
M. pusilla CCMP1545
M. pusilla RCC299
H. sapiens
O. lucimarinus
S. moellendorffii
P. patens (142)
P. patens (65)
P. vulgaris (1g)
P. vulgaris (7g)
P. sitchensis
M. domestica (157)
G. max (10g)
B. distachyon
O. sativa
P. virgatum
S. italica
S. bicolor
Z. mays
M. truncatula
G. max (3g)
G. max (19g)
C. sativus
M. guttatus
S. tuberosum
S. lycopersicum
P. trichocarpa (14g)
T. halophila
B. rapa
C. rubella
A. thaliana
A. lyrata
C. sinensis
C. clementina
P. trichocarpa (2g)
E. grandis
C. papaya
A. caerulea
G. raimondii (182)
T. cacao
G. raimondii (236)
M. domestica (193)
F. vesca
P. persica
R. communis
M. esculenta
C. subellipsoidea
V. carteri
C. reinhardtii
Supplemental Figure 1 cont.

## Slide 4
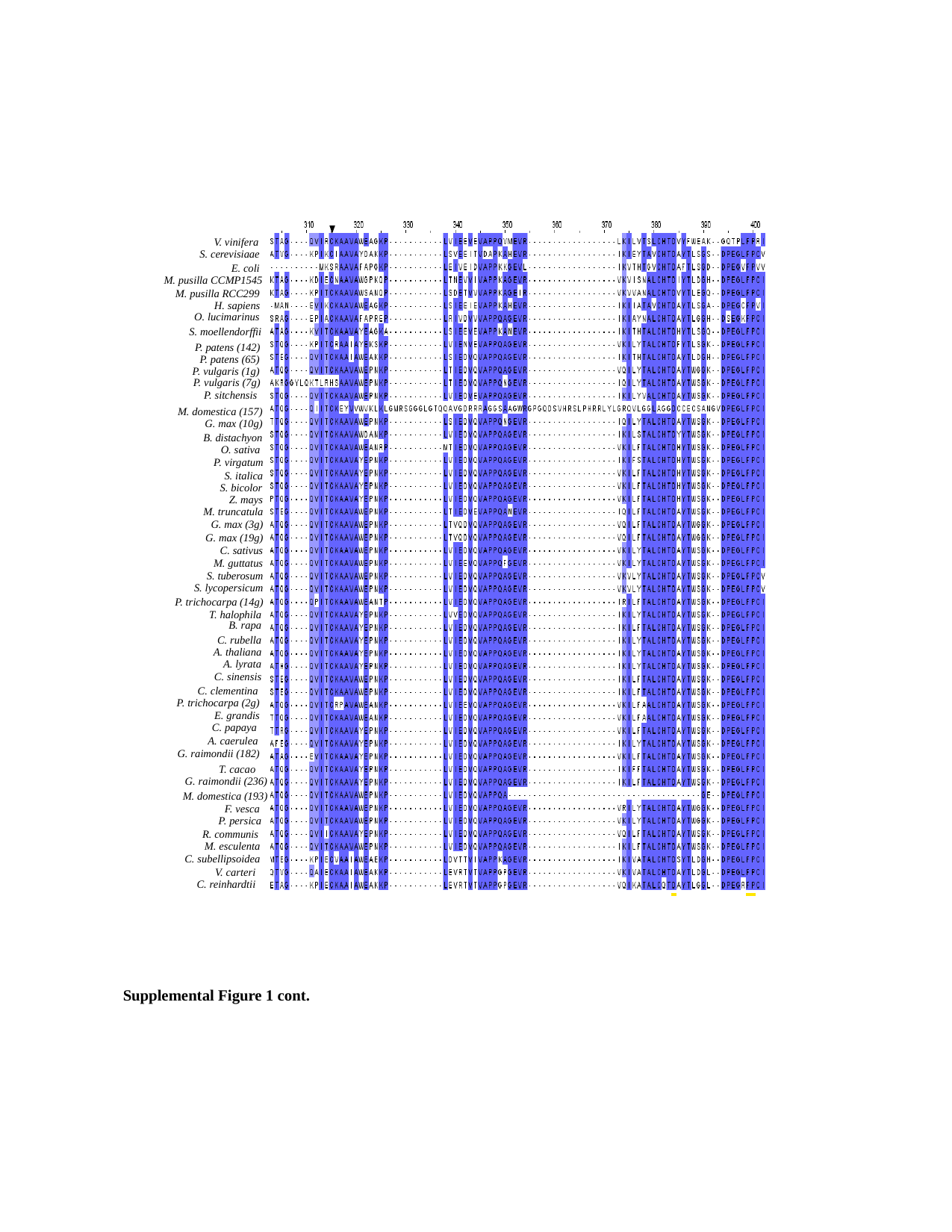

V. vinifera
S. cerevisiaae
E. coli
M. pusilla CCMP1545
M. pusilla RCC299
H. sapiens
O. lucimarinus
S. moellendorffii
P. patens (142)
P. patens (65)
P. vulgaris (1g)
P. vulgaris (7g)
P. sitchensis
M. domestica (157)
G. max (10g)
B. distachyon
O. sativa
P. virgatum
S. italica
S. bicolor
Z. mays
M. truncatula
G. max (3g)
G. max (19g)
C. sativus
M. guttatus
S. tuberosum
S. lycopersicum
P. trichocarpa (14g)
T. halophila
B. rapa
C. rubella
A. thaliana
A. lyrata
C. sinensis
C. clementina
P. trichocarpa (2g)
E. grandis
C. papaya
A. caerulea
G. raimondii (182)
T. cacao
G. raimondii (236)
M. domestica (193)
F. vesca
P. persica
R. communis
M. esculenta
C. subellipsoidea
V. carteri
C. reinhardtii
Supplemental Figure 1 cont.

## Slide 5
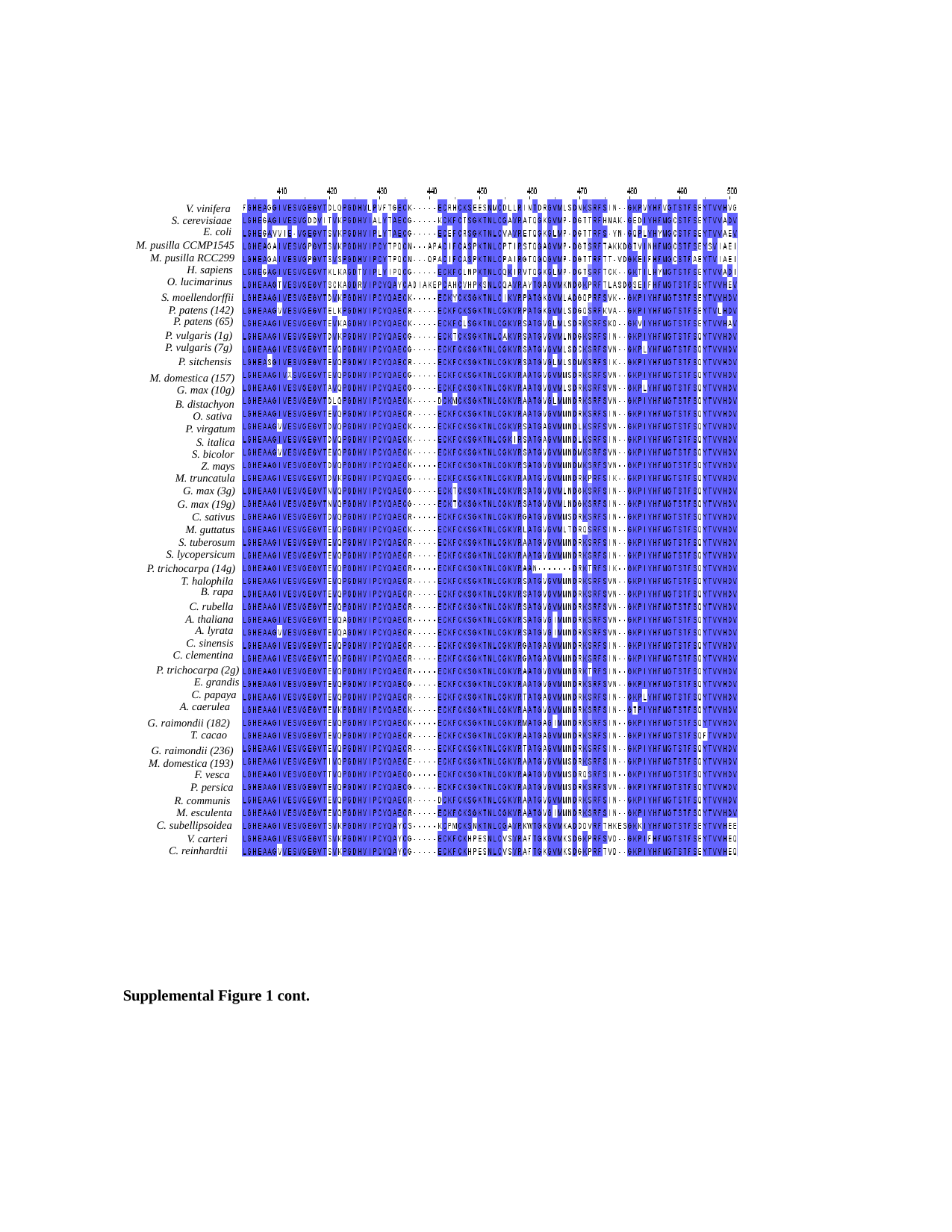

V. vinifera
S. cerevisiaae
E. coli
M. pusilla CCMP1545
M. pusilla RCC299
H. sapiens
O. lucimarinus
S. moellendorffii
P. patens (142)
P. patens (65)
P. vulgaris (1g)
P. vulgaris (7g)
P. sitchensis
M. domestica (157)
G. max (10g)
B. distachyon
O. sativa
P. virgatum
S. italica
S. bicolor
Z. mays
M. truncatula
G. max (3g)
G. max (19g)
C. sativus
M. guttatus
S. tuberosum
S. lycopersicum
P. trichocarpa (14g)
T. halophila
B. rapa
C. rubella
A. thaliana
A. lyrata
C. sinensis
C. clementina
P. trichocarpa (2g)
E. grandis
C. papaya
A. caerulea
G. raimondii (182)
T. cacao
G. raimondii (236)
M. domestica (193)
F. vesca
P. persica
R. communis
M. esculenta
C. subellipsoidea
V. carteri
C. reinhardtii
Supplemental Figure 1 cont.

## Slide 6
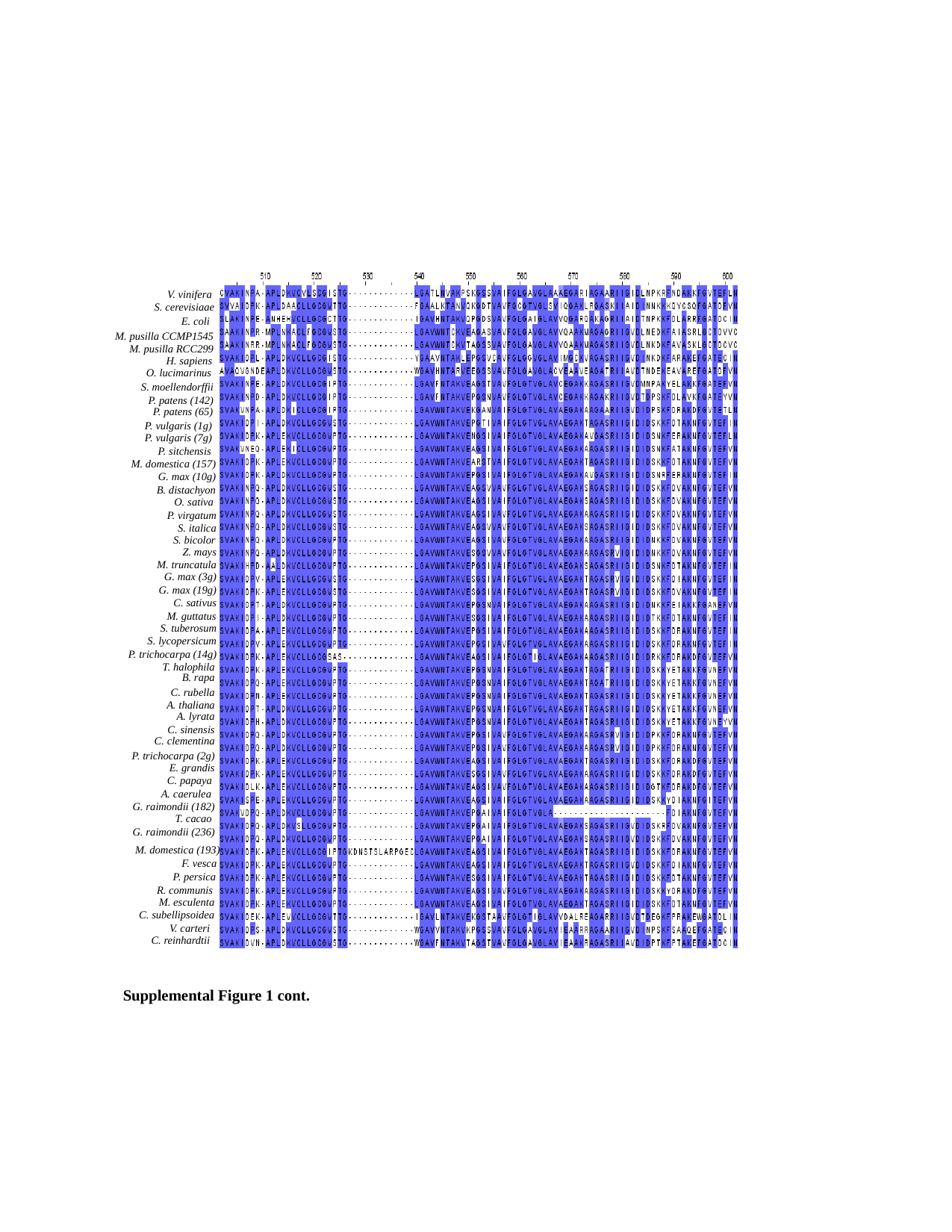

V. vinifera
S. cerevisiaae
E. coli
M. pusilla CCMP1545
M. pusilla RCC299
H. sapiens
O. lucimarinus
S. moellendorffii
P. patens (142)
P. patens (65)
P. vulgaris (1g)
P. vulgaris (7g)
P. sitchensis
M. domestica (157)
G. max (10g)
B. distachyon
O. sativa
P. virgatum
S. italica
S. bicolor
Z. mays
M. truncatula
G. max (3g)
G. max (19g)
C. sativus
M. guttatus
S. tuberosum
S. lycopersicum
P. trichocarpa (14g)
T. halophila
B. rapa
C. rubella
A. thaliana
A. lyrata
C. sinensis
C. clementina
P. trichocarpa (2g)
E. grandis
C. papaya
A. caerulea
G. raimondii (182)
T. cacao
G. raimondii (236)
M. domestica (193)
F. vesca
P. persica
R. communis
M. esculenta
C. subellipsoidea
V. carteri
C. reinhardtii
Supplemental Figure 1 cont.

## Slide 7
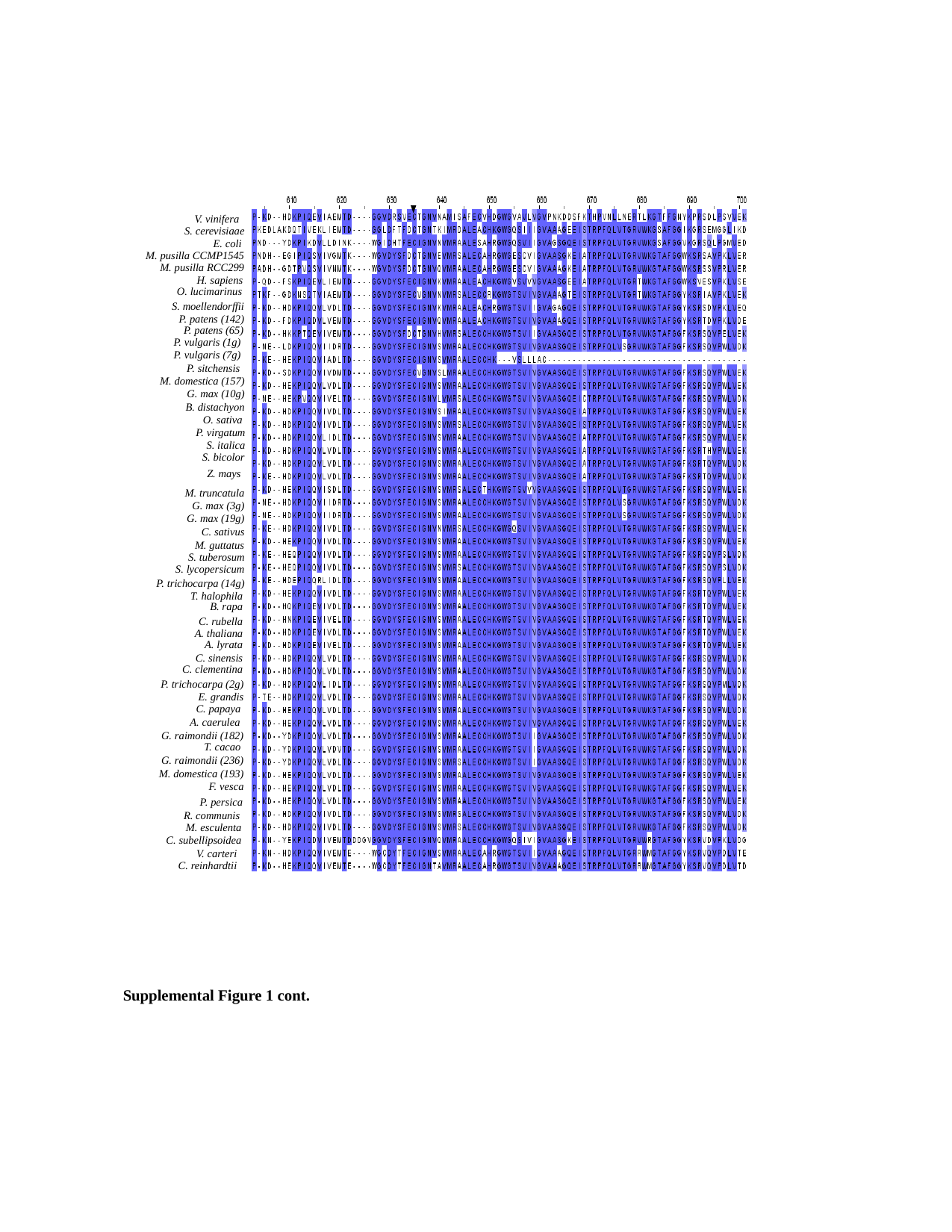

V. vinifera
S. cerevisiaae
E. coli
M. pusilla CCMP1545
M. pusilla RCC299
H. sapiens
O. lucimarinus
S. moellendorffii
P. patens (142)
P. patens (65)
P. vulgaris (1g)
P. vulgaris (7g)
P. sitchensis
M. domestica (157)
G. max (10g)
B. distachyon
O. sativa
P. virgatum
S. italica
S. bicolor
Z. mays
M. truncatula
G. max (3g)
G. max (19g)
C. sativus
M. guttatus
S. tuberosum
S. lycopersicum
P. trichocarpa (14g)
T. halophila
B. rapa
C. rubella
A. thaliana
A. lyrata
C. sinensis
C. clementina
P. trichocarpa (2g)
E. grandis
C. papaya
A. caerulea
G. raimondii (182)
T. cacao
G. raimondii (236)
M. domestica (193)
F. vesca
P. persica
R. communis
M. esculenta
C. subellipsoidea
V. carteri
C. reinhardtii
Supplemental Figure 1 cont.

## Slide 8
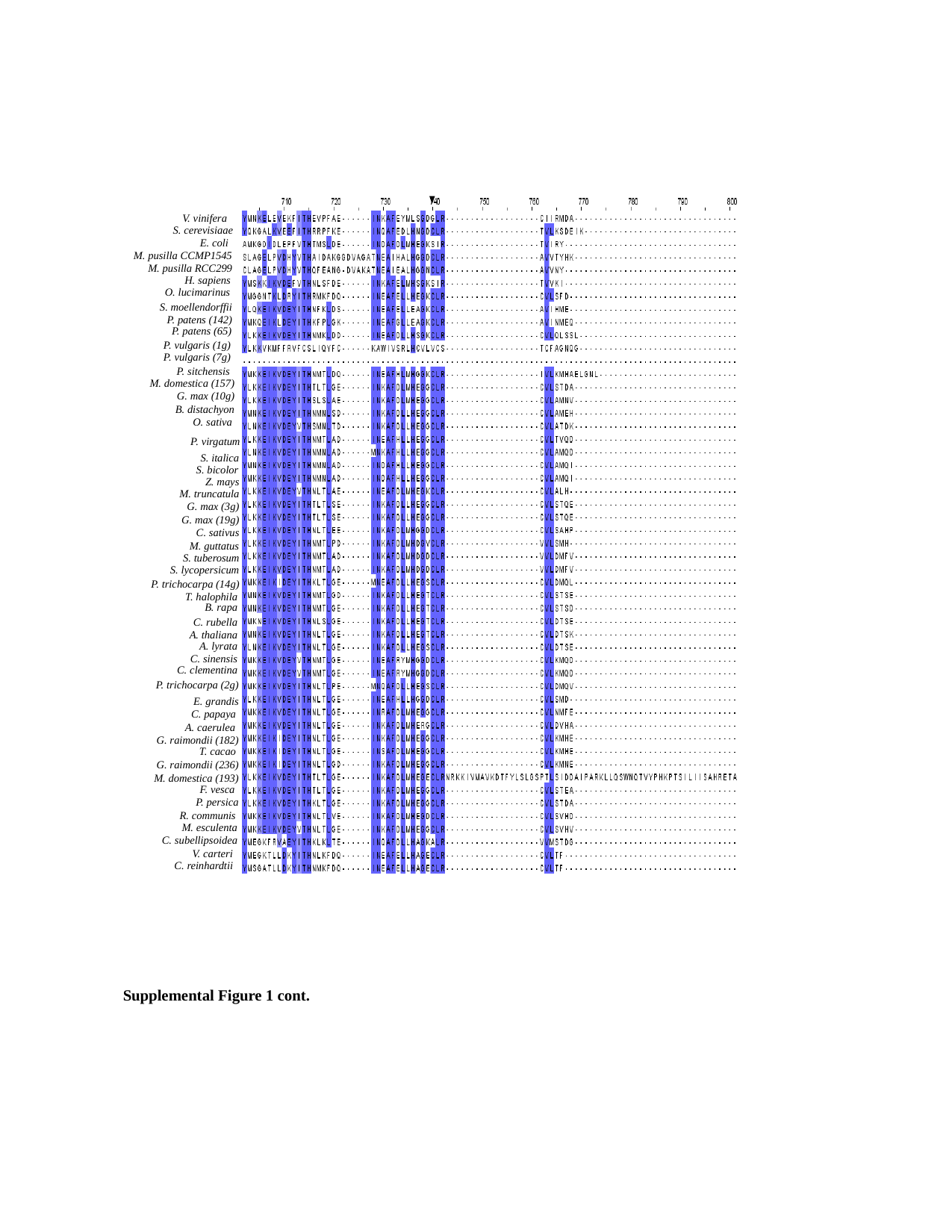

V. vinifera
S. cerevisiaae
E. coli
M. pusilla CCMP1545
M. pusilla RCC299
H. sapiens
O. lucimarinus
S. moellendorffii
P. patens (142)
P. patens (65)
P. vulgaris (1g)
P. vulgaris (7g)
P. sitchensis
M. domestica (157)
G. max (10g)
B. distachyon
O. sativa
P. virgatum
S. italica
S. bicolor
Z. mays
M. truncatula
G. max (3g)
G. max (19g)
C. sativus
M. guttatus
S. tuberosum
S. lycopersicum
P. trichocarpa (14g)
T. halophila
B. rapa
C. rubella
A. thaliana
A. lyrata
C. sinensis
C. clementina
P. trichocarpa (2g)
E. grandis
C. papaya
A. caerulea
G. raimondii (182)
T. cacao
G. raimondii (236)
M. domestica (193)
F. vesca
P. persica
R. communis
M. esculenta
C. subellipsoidea
V. carteri
C. reinhardtii
Supplemental Figure 1 cont.

## Slide 9
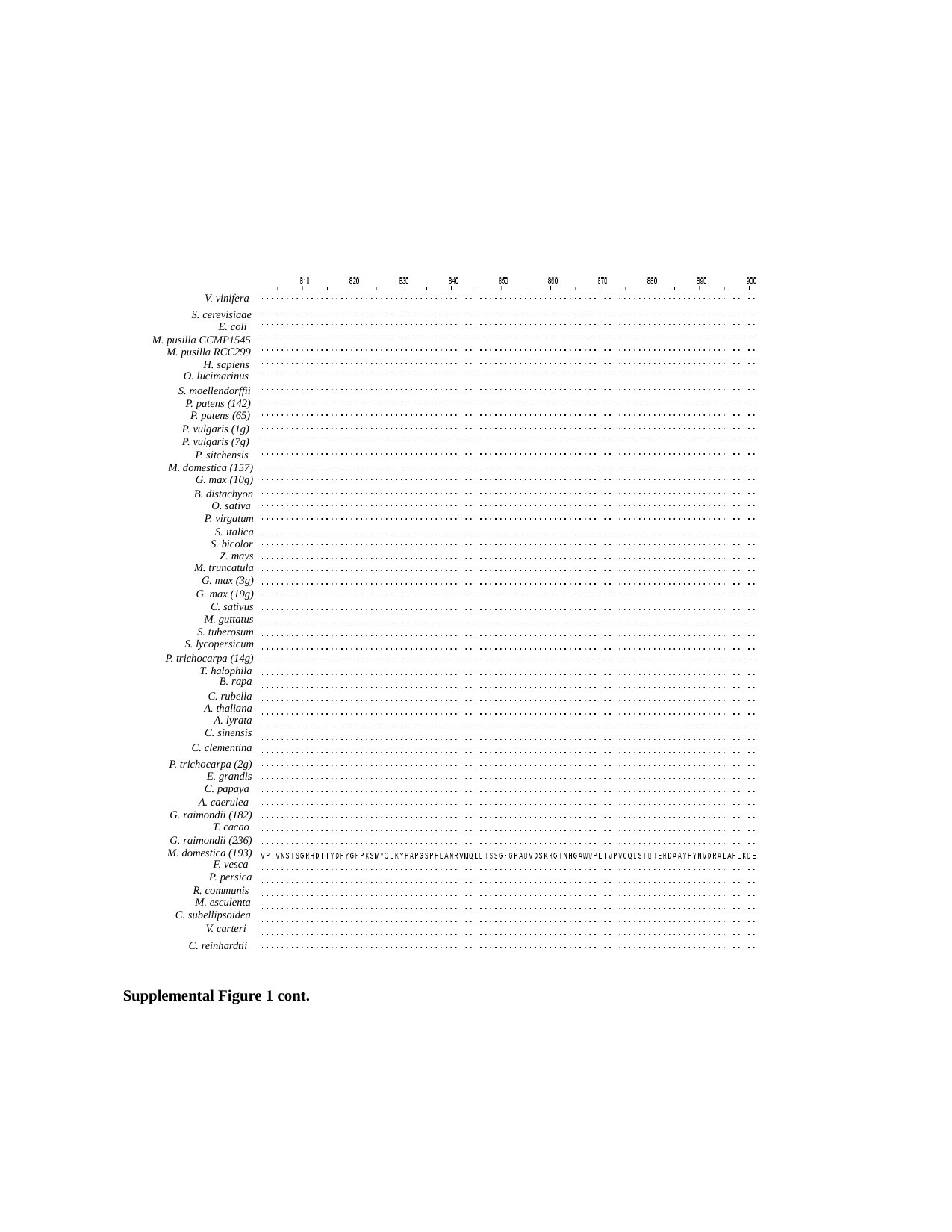

V. vinifera
S. cerevisiaae
E. coli
M. pusilla CCMP1545
M. pusilla RCC299
H. sapiens
O. lucimarinus
S. moellendorffii
P. patens (142)
P. patens (65)
P. vulgaris (1g)
P. vulgaris (7g)
P. sitchensis
M. domestica (157)
G. max (10g)
B. distachyon
O. sativa
P. virgatum
S. italica
S. bicolor
Z. mays
M. truncatula
G. max (3g)
G. max (19g)
C. sativus
M. guttatus
S. tuberosum
S. lycopersicum
P. trichocarpa (14g)
T. halophila
B. rapa
C. rubella
A. thaliana
A. lyrata
C. sinensis
C. clementina
P. trichocarpa (2g)
E. grandis
C. papaya
A. caerulea
G. raimondii (182)
T. cacao
G. raimondii (236)
M. domestica (193)
F. vesca
P. persica
R. communis
M. esculenta
C. subellipsoidea
V. carteri
C. reinhardtii
Supplemental Figure 1 cont.

## Slide 10
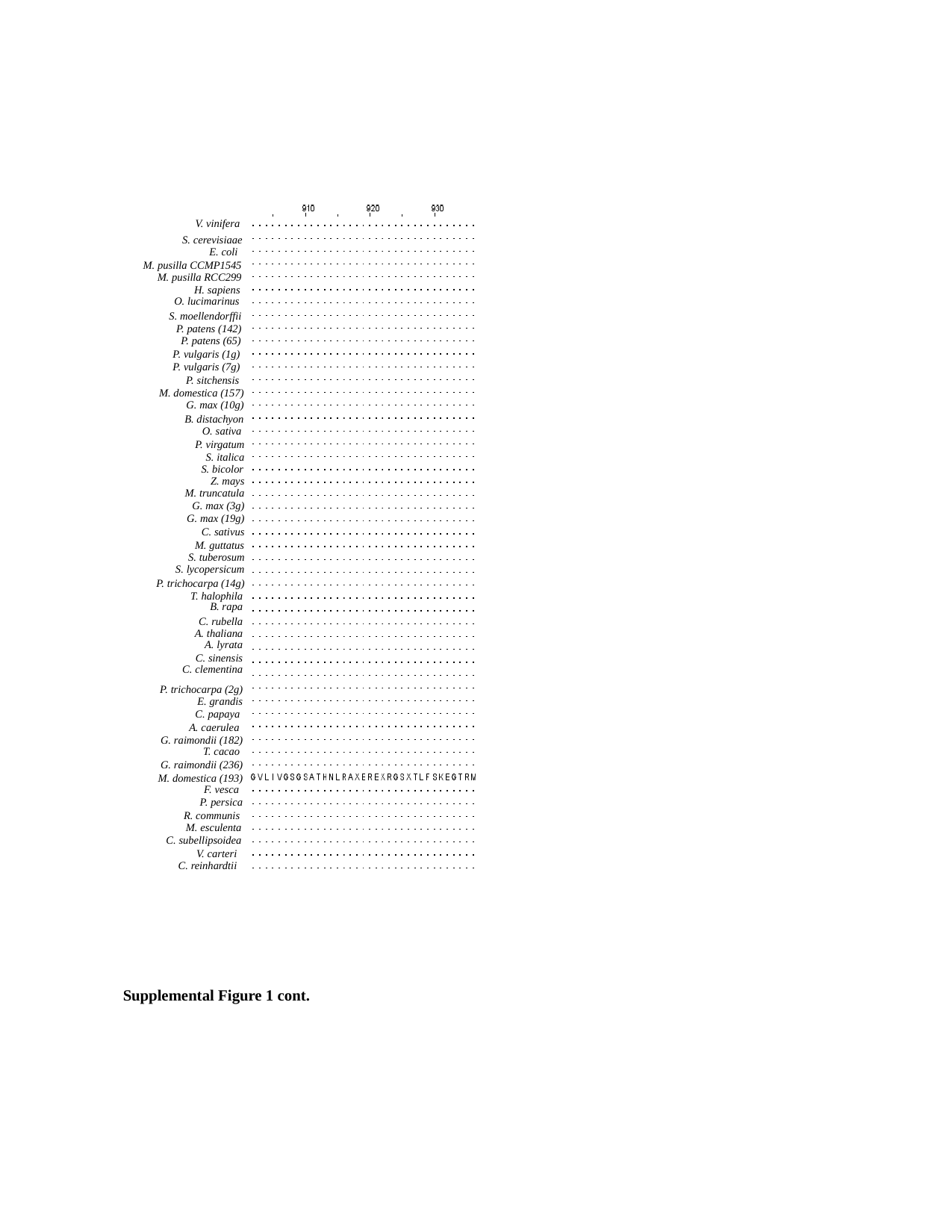

V. vinifera
S. cerevisiaae
E. coli
M. pusilla CCMP1545
M. pusilla RCC299
H. sapiens
O. lucimarinus
S. moellendorffii
P. patens (142)
P. patens (65)
P. vulgaris (1g)
P. vulgaris (7g)
P. sitchensis
M. domestica (157)
G. max (10g)
B. distachyon
O. sativa
P. virgatum
S. italica
S. bicolor
Z. mays
M. truncatula
G. max (3g)
G. max (19g)
C. sativus
M. guttatus
S. tuberosum
S. lycopersicum
P. trichocarpa (14g)
T. halophila
B. rapa
C. rubella
A. thaliana
A. lyrata
C. sinensis
C. clementina
P. trichocarpa (2g)
E. grandis
C. papaya
A. caerulea
G. raimondii (182)
T. cacao
G. raimondii (236)
M. domestica (193)
F. vesca
P. persica
R. communis
M. esculenta
C. subellipsoidea
V. carteri
C. reinhardtii
Supplemental Figure 1 cont.
